# Hommage à Jean-Paul Moreau (1936-2024)

**DOI:** 10.48327/mtsi.v5i2.2025.672

**Published:** 2025-04-16

**Authors:** Jean-Philippe CHIPPAUX

**Affiliations:** SFMTSI Société francophone de médecine tropicale et santé internationale (ancienne SPE), Institut Pasteur, 25 Rue du Dr Roux, 75015 Paris.

**Figure 1:**
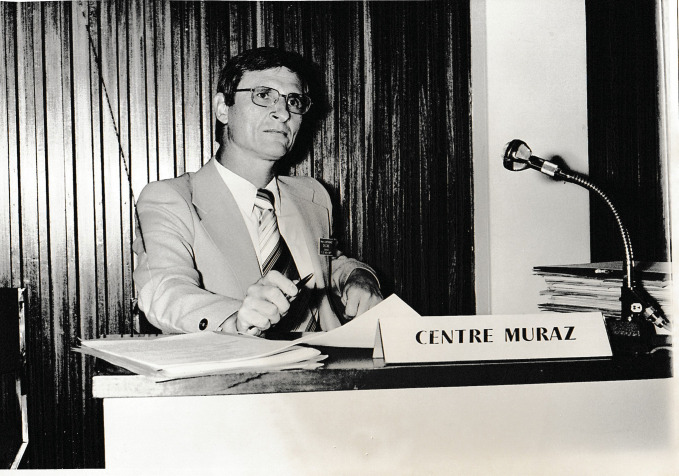
Jean-Paul Moreau au Centre Muraz de Bobo Dioulaso (crédit photo : archive familiale)

Jean-Paul Moreau nous a quitté le 22 novembre 2024 à l’âge de 88 ans.

Il est né à Lorient en 1936, dans une fratrie de 10 enfants, d'un père charpentier de marine et d'une mère employée des Postes. Il fit ses études primaires et secondaires sanctionnées par un baccalauréat littéraire. Attiré par la biologie humaine découverte lors de son année de terminale, il réussit le certificat de physique-chimie-biologie à Nantes et fit sa première année de médecine à l’École annexe du Service de santé de la marine à Brest. Il fut reçu au concours de l’École de Santé Navale à Bordeaux qu'il intégra en 1956. Il suivit les cours de l’École d'application du Pharo à Marseille en 1962. Son premier poste à la sortie du Pharo fut médecin-chef de la garnison de Majunga, à Madagascar.

À Tananarive, au cours des visites protocolaires incontournables pour un jeune médecin militaire, il fit la rencontre d'Edouard-Raoul Brygoo, alors directeur de l'Institut Pasteur de Madagascar, et d'André Dodin son sous-directeur. Cette rencontre et les relations qu'il entretint avec eux au cours de son séjour allait marquer la suite de sa carrière.

À Majunga, où il atterrit en juin 1963, Jean-Paul Moreau soignait les militaires de la garnison et leur famille. Il y fit la connaissance de Didier Ratsiraka, alors jeune enseigne de vaisseau du même âge que lui, et qui fut chef de l’État malgache à deux reprises, de 1975 à 1993, puis de 1997 à 2002. Parallèlement, Jean-Paul Moreau se forma à la microbiologie, encadré à distance par André Dodin. Il initia une recherche expérimentale sur la filariose de Bancroft. Celle-ci semblait absente de la région de Majunga alors que toutes les conditions pour sa transmission étaient réunies. Aidé par Guy Chauvet, entomologiste de l'ORSTOM^1^1Office de la recherche scientifique et technique d'outremer qui devint en 1998 l'Institut de recherche pour le développement, après s’être appelé quelques mois Institut français de recherche scientifique pour le développement en coopération., il mit en place un élevage de *Culex* qu'il infecta grâce à trois jeunes volontaires malgaches porteurs de microfilaires. Il démontra ainsi que le *Culex* local était réceptif et que les conditions pour que s'installe un foyer local de bancroftose étaient bien remplies. Jacques Prod'hon parasitologiste de l'ORSTOM, confirma quelques années plus tard la transmission locale de *Wuchereria bancrofti.* Jean-Paul Moreau identifia le mollusque hôte intermédiaire de *Schistosoma haematobium* et fit les premiers essais du niridazole chez l'humain. Les études immunologiques des petits bilharziens traités par le niridazole ont été menées avec la collaboration d'André Capron, alors volontaire du service national à l'Institut Pasteur de Madagascar. André Capron allait consacrer la suite de sa carrière au développement d'un vaccin contre les schistosomoses.

Au retour de Madagascar, Jean-Paul Moreau prépara le concours d'assistanat des hôpitaux militaires au Pharo. Reçu, il fit son premier semestre de stage au laboratoire de biologie médicale de l'hôpital Laveran, dirigé par Claude Chastel qui l'initia à la virologie. Son second semestre de stage s'est déroulé au Pharo dans le laboratoire des arbovirus. Encadré par Alain Chippaux, Jean-Paul Moreau traqua le virus du Nil occidental chez les moustiques camarguais.

Puis, il suivit le Grand Cours de l'Institut Pasteur l'année scolaire 1967-1968 à l'issue duquel il fut affecté à l'Institut de recherches médicales Louis Malardé de Papeete. Il fit équipe avec André Stanghellini sur la filariose lymphatique, retour à un amour de jeunesse, et avec Raymond Bagnis sur l'ichtyosarcotoxisme, notamment la ciguatera.

A l'occasion de plusieurs épidémies de dengue, notamment en 1969 et 1971, il étudia une trentaine de cas hémorragiques. Cette investigation soutenait la thèse de la sensibilisation des patients infectés auparavant par un autre sérotype viral. Jean-Paul Moreau passa le concours de spécialiste de biologie des hôpitaux des armées en 1973 et fut affecté à l'Institut Pasteur de Madagascar comme chef du laboratoire de biologie médicale auparavant dirigé par André Dodin. Il reprit ses recherches sur les schistosomoses en collaboration avec Pierre Coulanges. L’équipe mit au point une technique de sérodiagnostic de S. *mansoni* par immunofluorescence à l'aide de furcocercaires fixées sur lame. Plus simple et plus pratique que celle utilisant des coupes de ver adulte congelé, cette méthode présentait les mêmes performances. Faute de dépôt d'un brevet, elle fut commercialisée par Behring Werke l'année suivante… Dans le même temps, Jean-Paul Moreau poursuivait des essais thérapeutiques de différentes molécules contre les schistosomoses, la filariose lymphatique et divers parasites intestinaux. Il quitta Madagascar à la suite du coup d’état de 1975.

Après un cours passage au Pharo, au laboratoire des arbovirus, où il tenta sans succès de démontrer la transmission transovarienne du virus Tahyna chez *Aedes,* il fut affecté au Centre Muraz de Bobo Dioulaso en Haute Volta^2^2Aujourd'hui le Burkina Faso.. Il y resta de 1976 à 1981, supervisant les activités de surveillance et de recherche sur la trypanosomose, l'onchocercose, les schistosomoses, le paludisme, la fièvre jaune, les méningites, la poliomyélite.

En 1982 et 1983, Jean-Paul Moreau dirigea le laboratoire de l'Hôpital des armées de Metz. Il en profita pour se former à l'informatique et fit partie du chœur de l'Association lorraine des amis de la musique.

En juillet 1983, il fut nommé directeur de l'Institut Pasteur de Nouméa. Il y reprit avec le virologiste et entomologiste Pierre Fauran, les recherches sur la transmission transovarienne du virus de la dengue chez *Aedes.* Il développa les études sur l'hépatite B, les leptospiroses, la lèpre. La fin de son séjour fut marqué par les évènements de 1984-1988, notamment la tragédie d'Ouvéa (5 mai 1988). Il quitta Nouméa le 11 novembre 1988.

Il prit ensuite la direction de l'Institut Pasteur de la Guyane de 1988 à 1995. Après les lois de décentralisation de 1983 et 1985, les autorités départementales dénoncèrent la convention qui les liaient à l'Institut Pasteur de la Guyane.

Jean-Paul Moreau stabilisa la situation difficile et restructura l'Institut. Il rénova et mis aux normes les bâtiments historiques et pacifia le climat social. Il fit face à l’épidémie de dengue de 1991-1992 au cours de laquelle une centaine de cas hémorragiques a été observée. Il réorienta les activités du laboratoire d'entomologie. Celuici se focalisa, d'une part sur les vecteurs de la dengue et la sensibilité *d'Aedes aegypti* aux insecticides et, d'autre part sur la chimiorésistance de *Plasmodium falciparum*, notamment à la chloroquine. Le laboratoire supervisait, comme par le passé, les interventions de lutte antivectorielle menée par le service départemental de désinfection. Jean-Paul Moreau eu également le mérite d'organiser un nouvel axe de recherche concernant la rétrovirologie avec le VIH-sida, bien évidemment, mais aussi les infections aux virus HTLV.

Fin 1994, Jean-Paul Moreau prit sa retraite du Service de santé des armées avec le grade de médecin-chef des services (médecin général). Il fut alors recruté par l'Institut Pasteur et affecté comme directeur de l'Institut Pasteur de Dakar de 1995 à 2000. Le jour de sa prise de fonction, le 25 octobre 1995, il fut informé d'une épidémie de fièvre jaune dans la région de Kaffrine au centre du Sénégal. A l'occasion de cette épidémie, Didier Fontenille, entomologiste de l'ORSTOM et Jocelyn Thonnon, virologiste de l'Institut Pasteur, montrèrent que la transmission transovarienne du virus amaril lui permettait de se maintenir dans les œufs quiescents *d'Aedes* entre deux saisons des pluies. En juillet 1997, l'Institut Pasteur de Dakar perdit l'agrément de l'Organisation mondiale de la santé pour la vente de son vaccin amaril aux agences de l'Organisation des nations-unies. Il le retrouva un an plus tard…

Son séjour dakarois fut l'un de ceux qu'il apprécia le plus. Jean-Paul Moreau quitta l'Institut Pasteur de Dakar le 1^er^ juillet 2000 pour prendre sa retraite de l'Institut Pasteur.

Cependant, ses dernières années ne furent pas inactives. Érudit et curieux, Jean-Paul Moreau commença une nouvelle carrière d'historien des sociétés antiques au sein du département d'histoire de l'Université de Rennes 2. Il soutint en 2012 une maîtrise en histoire et sciences sociales des régions littorales et de la mer de l'Université de Bretagne Sud. Il s'intéressa au peuplement de Belle-Île-en-Mer par les Acadiens après la déportation dont ils firent l'objet après la guerre de 7 ans (1756-1763). Il montra que, contrairement à la légende, le quart de la population acadienne s’était intégré, tandis que le tiers avaient migré en Louisianne. Le reste s'est établi dans d'autres ports bretons.

Il publia, notamment, un triptyque sur l'histoire des religions publié chez L'Harmattan dans la collection Religions et Spiritualité^3^3« Disputes et conflits du christianisme : dans l'Empire romain et l'Occident médiéval », 2005; « L'anglicanisme : ses origines, ses conflits; du schisme d'Henri VIII à la bataille de la Boyne », 2006 et « Les avatars du protestantisme aux États-Unis de 1607 à 2007 », 2008.. Sérieusement documentés, ces ouvrages retracent les controverses théologiques et les guerres de religion menées par les chrétiens de l'an 30 à l'an 2007 et qui ont abouti au pluralisme confessionnel que nous connaissons aujourd'hui.

Il rencontra son épouse Armelle à Port-Louis dont elle était originaire. Ensemble pendant l’été, ils animaient des colonies de vacances « Boussac », ce qui les rapprocha. Enfant, la petite Armelle avait suivi ses parents dans les affectations ultra-marines de son père, notamment en Extrême-Orient. Sans le savoir, elle se préparait à sa future vie d’épouse de pastorien, organisant les nombreuses réceptions imposées par les fonctions de son mari. Elle a enseigné le français à Tahiti, en Afrique et Guyane. Ils avaient 4 enfants, 11 petits-enfants et 1 arrière-petit-fils.

Enjoué, parfois facétieux, Jean-Paul Moreau avait de l'humour, ce qui lui joua parfois des tours. Il était mélomane et, outre le chœur de musiques religieuses et classiques dont il fut membre lors de son séjour à Metz, il aimait entonner des chants bretons ou des chansons de variété.

Ces dernières années, retiré à Sarzeau sur la presqu’île de Rhuys, il fut un grand-père comblé… Jean-Paul Moreau a mené une carrière scientifique rigoureuse et éclectique, ce qui ne l'a pas empêché de restructurer certains des instituts qu'il a dirigés, lorsque cela était nécessaire, faisant souvent face à des situations politiques locales complexes.

Auteur de près d'une centaine de publications, Jean-Paul Moreau, était membre de la Société francophone de médecine tropicale et santé internationale (ex-Société de pathologie exotique) et de l'Association des anciens élèves de l'Institut Pasteur. Il a été président de la Société polymathique du Morbihan de 2009 à 2014.

Il était officier de la Légion d'honneur, officier de l'Ordre national du Mérite, détenteur de la Croix du combattant, Commandeur de l'Ordre national Malagasy; il a reçu la médaille d'honneur du Service de santé des armées, échelon argent, et la médaille d'or de l'Institut Pasteur.

Jean-Paul Moreau repose au cimetière de PortLouis aux côtés de sa femme Armelle, disparue le 3 juin 2024, et de leur fils Loeiz décédé le 5 octobre 1985 à l’âge de 22 ans.

**Figure 2:**
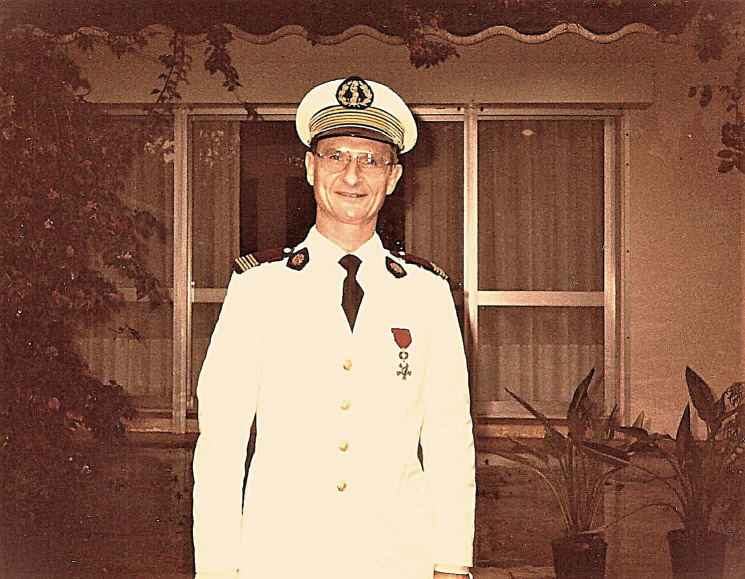
Jean-Paul Moreau le jour de sa réception dans l'Ordre de la Légion d'honneur (crédit photo : archive familiale)

## Remerciements

Je remercie Madame Ann-Gaëlle Moreau Paolillo de m'avoir rapporté quelques souvenirs de son père et de nous avoir confié les photographies qui illustrent cet hommage.
