# Supplementary material for: Tribute to Jean-Paul Moreau (1936-2024)
Source: Med Trop Sante Int. 2025 Apr 16;5(2):mtsi.v5i2.2025.672. [Article in French] doi: 10.48327/mtsi.v5i2.2025.672 (PMC12308578; doi:10.48327/mtsi.v5i2.2025.672)
Supplement: Related texts

# Research article: Tribute to Jean-Paul Moreau (1936-2024)

**Figure 1:** Jean-Paul Moreau at the Centre Muraz in Bobo Dioulaso (photo credit: family archive)

Jean-Paul Moreau died on November 22, 2024 at the age of 88.

He was born in Lorient in 1936, one of 10 children, whose father was a ship carpenter and whose mother worked for the post office. He completed his primary and secondary education, obtaining a baccalaureate in literature. Attracted by human biology, which he discovered during his final year of secondary school, he passed the Physics-Chemistry-Biology certificate in Nantes and did his first year of medicine at the Naval Medical School in Brest. He was accepted to the Naval Medical School in Bordeaux, which he entered in 1956. In 1962, he attended courses at the Ecole d'Application du Pharo in Marseilles. His first posting after leaving the Pharo was as medical officer to the garrison at Majunga, Madagascar. In Tananarive, during the formal visits that are essential for a young military doctor, he met Edouard-Raoul Brygoo, then director of the Pasteur Institute of Madagascar, and André Dodin, his deputy. This meeting and the relationships he maintained with them during his stay would mark the rest of his career.

In Majunga, where he arrived in June 1963, Jean-Paul Moreau treated the soldiers of the garrison and their families. There he met Didier Ratsiraka, then a young Sub-lieutenant of his own age, who was twice elected President of Madagascar, from 1975 to 1993 and from 1997 to 2002. At the same time, Jean-Paul Moreau trained in microbiology under the supervision of André Dodin. He initiated experimental research on Bancroft's filariasis. This disease seemed to be absent in the Majunga region, although all the conditions for its transmission were present. With the help of Guy Chauvet, an entomologist at ORSTOM^1^1Office de la recherche scientifique et technique outremer, which in 1998 became the Institut de recherche pour le développement (the French National Research Institute for Sustainable Development), after a few months as the Institut français de recherche scientifique pour le développement en coopération., he set up a *Culex* rearing program and infected the mosquitoes with the microfilariae of three young Madagascan volunteers. He demonstrated that the local *Culex* was receptive and that the conditions were in place for a local outbreak of bancroftosis. Jacques Prod'hon, a parasitologist at ORSTOM, confirmed the local transmission of *Wuchereria bancrofti* a few years later. Jean-Paul Moreau identified the mollusc intermediate host of *Schistosoma haematobium* and conducted the first human clinical trials of niridazole. The immunological studies on the young schistosomiasis patients treated with niridazole were carried out in collaboration with André Capron, then a national volunteer at the Pasteur Institute in Madagascar. André Capron would dedicate the rest of his career to developing a vaccine against schistosomiasis.

Upon his return from Madagascar, Jean-Paul Moreau studied at the Pharo to prepare for the competition to become an assistant in military hospitals. Once accepted, he spent the first six months of his training in the medical biology laboratory of the Laveran Hospital, under the direction of Claude Chastel, who introduced him to virology. His second six months of training took place at the Pharo in the arbovirus laboratory. Under the supervision of Alain Chippaux, Jean-Paul Moreau detected the West Nile virus in Camargue mosquitoes.

He then attended the Institut Pasteur Grand Cours during the 1967-1968 academic year, after which he was assigned to the Louis Malardé Institute of Medical Research in Papeete. He worked with André Stanghellini on lymphatic filariasis, a return to his childhood love, and with Raymond Bagnis on ichthyosarcotoxism, particularly ciguatera. During several dengue epidemics, particularly in 1969 and 1971, he studied about thirty hemorrhagic cases. This study supported the theory of sensitization of patients previously infected with another viral serotype.

In 1973, Jean-Paul Moreau qualified as a specialist in biology for military hospitals and was assigned to the Institut Pasteur in Madagascar as head of the medical biology laboratory previously directed by André Dodin. He resumed his research on schistosomiasis in collaboration with Pierre Coulanges. The team developed a serodiagnostic technique for S. *mansoni* by immunofluorescence using furcocercariae fixed on a microscope slide. This method was simpler and more practical than the method using frozen sections of adult worms, and performed equally well. In the absence of a patent application, it was commercialized by Behring Werke the following year. At the same time, Jean-Paul Moreau was conducting therapeutic trials of various molecules against schistosomiasis, lymphatic filariasis and various intestinal parasites. He left Madagascar after the 1975 coup.

After a brief stint at Pharo in the Arbovirus Laboratory, where he unsuccessfully attempted to demonstrate transovarian transmission of Tahyna virus in *Aedes,* he was assigned to the Muraz Center at Bobo Dioulasso in Upper Volta^2^2Today, Burkina Faso.. He remained there from 1976 to 1981, supervising surveillance and research activities on trypanosomiasis, onchocerciasis, schistosomiasis, malaria, yellow fever, meningitis, poliomyelitis, etc.

In 1982 and 1983, Jean-Paul Moreau directed the laboratory of the military hospital in Metz. He took the opportunity to train in computer science and was a member of the choir of the Association lorraine des amis de la musique.

In July 1983, he was appointed director of the Institut Pasteur in Nouméa. There, with the virologist and entomologist Pierre Fauran, he resumed research on the transovarian transmission of the dengue virus in *Aedes*. He developed studies on hepatitis B, leptospirosis and leprosy. The end of his stay was marked by the events of 1984-1988, in particular the tragedy of Ouvéa (May 5, 1988). He left Nouméa on November 11, 1988.

He then became director of the Institut Pasteur in French Guiana from 1988 to 1995. Following the decentralization laws of 1983 and 1985, the departmental authorities terminated the agreement between themselves and the Institut Pasteur in French Guiana. Jean-Paul Moreau stabilized the difficult situation and restructured the Institute. He renovated the historic buildings and brought them up to standard, and he also improved the social climate. He dealt with the dengue epidemic of 1991-1992, during which about a hundred hemorrhagic cases were observed. He redirected the activities of the entomology laboratory. The laboratory focused on dengue vectors and the sensitivity of *Aedes aegypti* to insecticides, as well as the chemoresistance of *Plasmodium falciparum,* particularly to chloroquine. As in the past, the laboratory monitored the vector control activities carried out by the departmental disinfection service. Jean-Paul Moreau also had the merit of organizing a new line of research concerning retrovirology, with HIV-AIDS of course, but also HTLV virus infections.

At the end of 1994, Jean-Paul Moreau retired from the Armed Forces Health Service with the rank of Chief Medical Officer (Major General). He was then recruited by the Institut Pasteur and appointed Director of the Institut Pasteur in Dakar from 1995 to 2000. On the day he began his assignment, October 25, 1995, he was informed of an epidemic of yellow fever in the Kaffrine region of central Senegal. During this epidemic, Didier Fontenille, an entomologist at ORSTOM, and Jocelyn Thonnon, a virologist at the Institut Pasteur, showed that transovarian transmission of the yellow fever virus allowed it to survive in the dormant eggs of the *Aedes* mosquito between the rainy seasons. In July 1997, the Institut Pasteur in Dakar lost World Health Organization approval to sell its yellow fever vaccine to United Nations agencies. It regained it a year later…

His time in Dakar was one of his happiest. Jean-Paul Moreau left the Institut Pasteur in Dakar on July 1, 2000 to retire.

However, his last years were not idle. Erudite and curious, Jean-Paul Moreau began a new career as a historian of ancient societies at the History Department of Rennes 2 University. In 2012, he obtained a Master's degree in History and Social Sciences of Coastal Regions and the Sea from the University of Southern Brittany. He was interested in the settlement of Belle-Îleen-Mer by the Acadians after their deportation following the Seven Years’ War (1756-1763). He showed that, contrary to legend, a quarter of the Acadian population had integrated, while a third had migrated to Louisiana. The rest settled in other Brittany ports.

In particular, he published a triptych on the history of religions, published by L'Harmattan editor in the collection Religions et Spiritualité^3^3*Disputes et conflits du christianisme : dans l'Empire romain et l'Occident medieval,* 2005; *L'anglicanisme : ses origines, ses conflits; du schisme d'Henri VIII à la bataille de la Boyne*, 2006 and *Les avatars du protestantisme aux Etats-Unis de 1607 à 2007,* 2008.. These works, which are seriously documented, trace the theological controversies and religious wars waged by Christians from the year 30 to the year 2007, which led to the confessional pluralism we know today.

He met his wife, Armelle, in Port-Louis, where she was from. They spent summers together at the “Boussac” summer camps, which brought them closer together. As a child, little Armelle had followed her parents on her father's overseas postings, especially in the Far East. Unbeknownst to her, she was preparing for her future life as a pastorian's wife, organizing the numerous receptions required by her husband's duties. She taught French in Tahiti, Africa and Guyana. They had 4 children, 11 grandchildren and 1 great-grandchild

Cheerful, sometimes facetious, Jean-Paul Moreau had a sense of humor that sometimes played tricks on him. He was a music lover and in addition to the religious and classical choir of which he was a member during his stay in Metz, he liked to sing Brittany songs or pop songs.

In recent years, retired in Sarzeau on the Rhuys peninsula, he was a very happy grandfather… Jean-Paul Moreau had a rigorous and eclectic scientific career, which did not prevent him from restructuring, when necessary, some of the institutes he directed, often dealing with complex local political situations.

Author of nearly a hundred publications, Jean-Paul Moreau was a member of the Société francophone de médecine tropicale et santé internationale (formerly the Société de pathologie exotique) and the Association des anciens élèves de l'Institut Pasteur. He was President of the Polymathic Society of Morbihan from 2009 to 2014.

He was an officer of the Légion d'honneur, an officer of the Ordre national du Mérite, holder of the Croix du combattant, Commander of the Ordre national Malagasy; he received the silver medal of honor of the Army Health Service and the gold medal of the Institut Pasteur.

Jean-Paul Moreau is buried in the cemetery of Port-Louis alongside his wife Armelle, who died on June 3, 2024, and their son Loeiz, who died on October 5, 1985 at the age of 22.

**Figure 2:** Jean-Paul Moreau on the day of his induction into the Order of the Légion d'honneur (photo credit: family archive)

## Acknowledgements

I would like to thank Mrs. Ann-Gaëlle Moreau Paolillo for sharing some of her father's memories with me and for entrusting us with the photographs that illustrate this tribute.
